# Introducing a New Experimental Islet Transplantation Model using Biomimetic Hydrogel and a Simple High Yield Islet Isolation Technique

**DOI:** 10.18869/acadpub.ibj.21.4.218

**Published:** 2017-07

**Authors:** Jamal Mohammadi Ayenehdeh, Bahareh Niknam, Seyed Mahmoud Hashemi, Hossein Rahavi, Nima Rezaei, Masoud Soleimani, Nader Tajik

**Affiliations:** 1 Department of Immunology, School of Medicine, Tehran University of Medical Sciences, Tehran, Iran; 2 Immunology Research Center (IRC), Iran University of Medical Sciences, Tehran, Iran; 3 Department of Immunology, School of Public Health, Tehran University of Medical Sciences, Tehran, Iran; 4 Department of Immunology, School of Medicine, Tehran, Shahid Beheshti University of Medical Sciences, Iran; 5 Research Center for Immunodeficiencies, Tehran University of Medical Sciences, Children’s Medical Center, Tehran, Iran; 6 Universal Scientific Education and Research Network (USERN), Tehran, Iran; 7 Department of Stem Cell Biology, Stem Cell Technology Research Center, Tehran, Iran

**Keywords:** Islet pancreas, Isolation, Transplantation, Type 1 diabetes mellitus

## Abstract

**Background:**

Islet transplantation could be an ideal alternative treatment to insulin therapy for type 1 diabetes Mellitus (T1DM). This clinical and experimental field requires a model that covers problems such as requiring a large number of functional and viable islets, the optimal transplantation site, and the prevention of islet dispersion. Hence, the methods of choice for isolation of functional islets and transplantation are crucial.

**Methods:**

The present study has introduced an experimental model that overcomes some critical issues in islet transplantation, including *in situ* pancreas perfusion by digestive enzymes through common bile duct. In comparison with conventional methods, we inflated the pancreas in Petri dishes with only 1 ml collagenase type XI solution, which was followed by hand-picking isolation or Ficoll gradient separation to purify the islets. Then we used a hydrogel composite in which the islets were embedded and transplanted into the peritoneal cavity of the streptozotocin-induced diabetic C57BL/6 mice.

**Results:**

As compared to the yield of the classical methods, in our modified technique, the mean yield of isolation was about 130-200 viable islets/mouse pancreas. *In vitro* glucose-mediated insulin secretion assay indicated an appropriate response in isolated islets. In addition, data from *in vivo* experiments revealed that the allograft remarkably maintained blood glucose levels under 400 mg/dl and hydrogel composite prevents the passage of immune cells.

**Conclusion:**

In the model presented here, the rapid islet isolation technique and the application of biomimetic hydrogel wrapping of islets could facilitate islet transplantation procedures.

## INTRODUCTION

Type 1 diabetes mellitus (T1DM) is characterized as a T-cell-mediated autoimmune disease in which immune destruction of pancreatic β cells progressively leads to hyperglycemia[1-4]. Diabetes is rapidly growing worldwide, and published data have indicated that it is the third most common chronic disease among American youth. The health-related cost of the disease has been estimated to be more than 10 billion dollars annually[5-8].

Pancreas transplantation is considered as a therapeutic choice[9]. The first option is transplanting the whole pancreas from a cadaveric donor; however, the shortage of cadaveric donors and the requirement of life-long multiple immunosuppressive drugs plus hyperinsulinemia necessitate searching alternative cell sources[10]. The whole pancreas transplantation as a major surgery causes a greater risk of complications and even death.

Insulin-producing β cells along with other cells involve in glycemic regulation and reside in the pancreatic islets of Langerhans[11]. Due to the lack of extra cells and less invasive operation, it is believed that the islet transplantation is an ideal alternative to whole pancreas transplantation[12-14]. As an initial step, it is required to have a large number of functional and viable islets. Successful isolation of the functional islets and the method of transplantation are very essential for their application in diabetes-related studies[1]. Islets are purified by techniques such as hand-picking isolation (HPI), cell cultivation, filtration, and Ficoll gradient separation (FGS). Each method has its own disadvantages. For example, the HPI method is not convenient for those experiments that need a large number of islets. Cultivation of islets is also a time-consuming procedure. In addition, islets purification by filtration is not an accurate strategy due to islet size heterogeneity. FGS depends on differences in cell density between islets (~1.059 g/ml) and exocrine cells (1.059-1.074 g/ml). FGS has been considered as the most favorable approach but it has toxic effects on islets[15,16]. Pancreas digestion for islet isolation is commonly carried out by an enzymatic process and often is followed by purifying via HPI or FGS.

Considering the above mentioned points, most of the islet isolation procedures are expensive as they require complicated manipulation of pancreas perfusion through common bile duct along with dispensable tissue digestion by the high volume of the enzyme. Furthermore, isolated islets are purified by density gradient centrifugation in multiple toxic density gradient components or hand-pick up under a microscope[17].

There are several factors that contribute in successful dissociation of pancreas and isolation of islets. These factors include the quality and the optimal concentration of digestive enzyme, the duration of enzyme digestion, and the experience of operating technicians. Regarding these criteria, we herein present a reliable, rapid and cost-effective method to obtain viable and functional mouse islets for *in vitro* and *in vivo* studies. The other important issue is the optimal transplantation site.

Many studies have been conducted to find a suitable site for islet transplantation. In experimental animal models of diabetes, various sites have been studied for islet transplantation such as the peritoneum, liver via the portal vein, blood vessels, muscle, bone marrow, omental pouch, or renal capsule[18]. The most common sites for transplantation are renal capsule in rodents, as well as liver via the portal vein in human. However, the infusion of islets *via* portal vein has various problems such as autoimmune destruction of the graft and thrombosis in the liver[19].

The dispersion of islets into the transplantation site is another challenge in this procedure. Kerby *et al*.[20] have used alginate compounds for islet micro-encapsulation that wraps every islet and transplants them into the peritoneum disperse into peritoneal cavity. Another problem is the loss of the entire structure of the islets and decomposing them into the single cells in the transplantation site. To overcome these obstacles, it is necessary to introduce a model to diminish dispersion and decompose the islets. In our model, we embedded the allograft islets into the hydrogel in a tissue-like manner and gathered them in close contact with each other to form the hydrogel composite. It is very suitable for further assessment such as direct effects of the additional agents (e.g. feeder or nursing cells) on the islets. The pore size of the hydrogel could be controlled by adjusting ingredients concentration to avoid the hydrogel cell output or immune cell entrance.

Consequently, the hydrogel makes a guard then protects the islets from future attack by the immune cells. Due to the large size of our hydrogel transplant, peritoneum cavity is better site than the others. On the other hand, it is very accessible and more suitable than the other sites in human transplantation. Therefore, this model presents a rapid islet isolation technique and introduces the application of a biomimetic hydrogel wrapping of islets, which may facilitate islet transplantation procedures. This system can also be applicable to experimental and clinical treatments as a delivery system for manipulated cells or certain drugs.

## MATERIALS AND METHODS

### Experimental animals

The transplant was between two different strains of mice, BALB/c and C57BL/6 as a donor and recipient, respectively. The mice were at 6-8 weeks of age and obtained from Pasteur Institute of Iran (Tehran). The animals were housed on a 12-h light/dark cycle with free access to food and water and euthanized according to the Animal Care guidelines of Tehran University of Medical Sciences (TUMS), Iran. The mice were then acclimatized to the new situation for a week before the experiments.

### Diabetes induction protocol using low-dose streptozotocin (STZ)

The C57BL/6 mice were rendered diabetic by Animal Models of Diabetic Complications Consortium (AMDCC) Protocols. Multiple low-doses (50 mg/kg, for 5 consecutive days) of STZ (Sigma, USA) were solubilized in the sodium citrate buffer, pH 4.5, which was injected intraperitoneally within 10 minutes of preparation. The mice were supplied with 10% sucrose water to avoid sudden hypoglycemia post injection. The STZ-Na Citrate buffer solution was injected during 20 minutes from its preparation because STZ degrades in the Na-Citrate buffer. The mice were tested for serum insulin and for sufficient levels of hyperglycemia at four weeks post-injection.

### Isolation of the islets from the mouse pancreas

To improve yields and viability of isolated islets, numerous modifications such as minimizing the enzymatic digestion period and optimizing enzyme concentration were examined. The entire procedure was carried out under aseptic conditions as follows:

### Pancreas removal and inflation

**Fig. 1:**
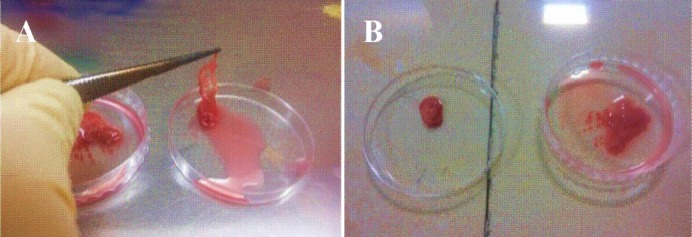
The inflation of the pancreas. Inflated pancreases are shown in both Petri dishes. Its swollen appearance was seen (A). Inflated pancreas is shown next to a freshly isolated pancreas (B). The inflated pancreas is approximately five fold larger than the freshly isolated ones.

After anesthetizing the mice with intraperitoneal injection of ketamine (10 mg/kg) and xylazine (100 mg/kg) mixture, and the middle abdominal skin was opened via a V-shape incision. To inflate the pancreas, the muscular layer in abdominal cavity must be opened, and the spleen is used as a holder to fix *in situ*. The pancreas and the spleen were carefully excised and pulled away from the intestines, stomach, and liver and placed in 100-mm Petri dishes, and then the spleen was removed. Afterwards, the mice were euthanized by cervical dislocation. The pancreas was held with a forceps and inflated with 1 ml cold collagenase type XI (1 mg/ml, Sigma, Germany) solution, containing RPMI 1640 medium and CaCl_2_ at least 10 times from several sides using a 30½ inch needle (bent at a 45° angle). The swollen appearance of pancreas is illustrated in Figure 1A and 1B. Infusion time did not exceed five minutes. It should also be noted that the pancreas perfusion was carried out *in situ* or immediately after removal from anesthetized mice and did not take more than five minutes. If perfusion needed more time, it was performed on ice. Inflated pancreas was transferred to a 15-ml conical tube and incubated at 37°C for 10 minutes until the separation of islets from the acinar tissue. The tube was shaken gently two or three times during the incubation period to disrupt the disintegrating tissue. After incubation, the digested material was filtered through pre-wetted filter (420 µm pore size) transferred to a 15-ml conical tube by which its large pieces were separated from the digested tissue (Tube #1). Then the strainer was inverted into a Petri dish to mince the large pieces of pancreas into approximately 1 mm^3^ pieces and returned to the 15-ml conical tube (Tube 2). Tube #1 was centrifuged at 250 ×g for 5 minutes, and the supernatant was replaced with 10-15 ml cold PBS to terminate enzymatic digestion. The islets were then purified from the prepared suspension. The aspirated supernatant containing digestion enzyme was transferred to Tube #2 containing the large pieces of tissue for further digestion. Tube #2 was incubated at 37°C for 10 minutes under continuous agitation to disrupt the undigested pieces of pancreas until the suspension turned homogeneous. Once dissolved in very fine particles, the tissue suspension was filtered (420 µm pore size strainer), and the digestion was stopped as previously described. The suspension was then ready for islets purification. In the steps of this protocol, depending on the available resources and expertise, the following two methods were followed to purify the islets.

### Hand-picking isolation

**Fig. 2:**
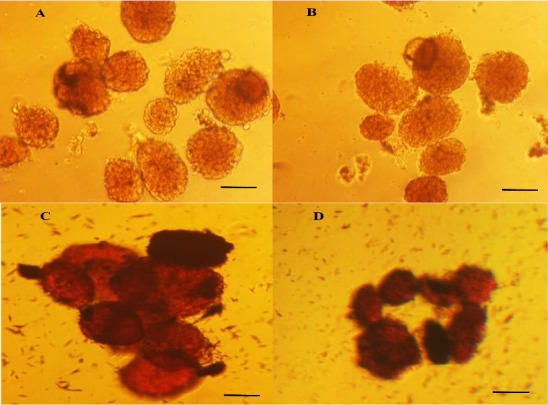
Isolated mouse pancreatic islets. The islets were obtained by two methods of hand-picking (A and C) and Ficoll separation (B and D). The parts of C and D show the same islets after dithizone staining. Images were acquired on an inverted microscope at 40× magnification. Scale bars represent 200 μm.

In this procedure, the contents of both tubes were transferred to a sterile Petri dish, and the islets were manually picked using a 10-µl sterile sampler under a stereomicroscope in a laminar hood (Fig. 2A).

### Ficoll gradient separation

The contents of both tubes were transferred to a 50-ml conical tube. Then the following steps were continued by centrifugation at 900 ×g for 5 minutes, and the supernatant was discarded. The pellet was washed three times with 10-ml cold Hank’s buffer, and the resulting pellet was resuspended in a 15-ml RPMI 1640 medium containing 11 mM glucose supplemented with 10% fetal calf serum, 100 units/ml penicillin, and 0.1 mg/ml streptomycin sulfate (Sigma, Germany). It was then filtered through a pre-wetted 70 µm cell strainer, which was inverted over a 15-ml conical tube. Next, the captured islets were rinsed with 15-ml BSA-free RPMI 1640. In order to prepare a discontinuous density gradient, 10-ml Ficoll (Sigma-Aldrich) density 1100[21] (50 ml of 1.077 g/cm^3^[22] and 60 ml of 1.119 g/cm^3^) was slowly added over the islets suspension and incubated in the dark at 4°C for 1 h. The gradient was spun at 200 ×g at 4°C for 20 minutes, and the upper layer was collected. The islets were returned to a 50-ml conical tube containing 25 ml cold Hank’s buffer (supplemented by 10% BSA) and centrifuged at 250 ×g for 5 minutes. The pancreatic islets were sedimented in the resulting solution (Fig. 2B).

### Islet culture and count

The islets were cultured in RPMI 1640 medium (11 mM glucose, supplemented with 10% fetal calf serum, 100 units/ml penicillin, and 0.1 mg/ml streptomycin sulfate, Sigma, Germany) in a humidified atmosphere of 5% CO_2_ at 37°C until transplantation. In aqueous environments, the islets tend to attach to each other so that manual counting becomes difficult. Thus, for partial detachment, the islets were treated with trypsin/EDTA at 37°C for 15-20 minutes and then centrifuged at 250 ×g at 4°C for 10 minutes. The pellet was resuspended in Hanks’ balanced salt solution and quantified in duplicate using an islet standard diameter of about 150 µm under an inverted microscope (4× objective lens). Islets smaller than 50 μm (maximum diameter) were excluded from the manual count[23].

### Cell viability assay

The viability of the purified islets were analyzed by a method based on the dye exclusion assay described by Altman *et al*.[24]. In this protocol, unlike the dead cells that are stained in blue, viable cells are not stained and are able to exclude Trypan blue dye. An equal volume of islet suspension containing 20 islets was obtained from each method and was treated with Trypan blue solution with a final concentration of 0.2 mg/ml at room temperature after incubation for 3-5 minutes. The ratio of viable to non-viable cells were checked under an inverted microscope[25].

### Dithizone staining of islets

The purified islets were counted and characterized by dithizone staining as shown in Figure 2C and 2D. Dithizone is a zinc-chelating agent that stains insulin within the islets, producing a crimson red color[26]. This color clearly can differentiate pancreatic islets from its acinar cells. Because of the toxic effects, the islets must be immediately washed with PBS, if used for transplantation or functional assays.

### Hydrogel preparation

For preparation of the 100 µl hydrogel (3D-Life Dextran-PEG Hydrogel kit, Cellendes, Germany), distilled water, 10× concentration buffer (pH 5.5), and maleimide-dextran were mixed in a reaction tube. After 5 minutes, 100 islets were added to the mixture. Polyethylene glycols (PEG-Link) were placed on the surface of a culture dish, and the content of the reaction tube was aspirated into a pipette tip and mixed with the PEG-Link by gentle pipetting. After at least one minute that the gel was formed, the culture medium was carefully added to cover the gel and incubated at 37°C until transplantation.

### Insulin secretion

For insulin secretion assay, it is necessary to culture and count the isolated islets, as previously described. Care was taken to isolate the islets under aseptic conditions and use sterile tubes and flasks. Equal numbers of similar sized islets were purified by HPI or FGS, as described above, and washed in PBS (8 g NaCL, 0.2 g KCL, 1.5 g NaHPO_4_, 0.2 g KH_2_PO4, pH 7.3) and grouped separately. Triplicate batches of 10 islet equivalents well were obtained from each method and divided into three groups: T0 (control group), T1 (150 mg/dl glucose), and T2 (300 mg/dl glucose) and preincubated in 2 ml of control medium (RPMI 1640+10% FBS+1% glutamine+1% penicillin/ streptomycin), pH 7.4, at 95% O_2_ and 5% CO_2_ at 37°C for 30 minutes. After the preincubation period, the first well from each batch was considered as a control medium well (T0) and the other wells (T1 or T2) as test medium wells. Glucose solution was added to the test medium wells at a final concentration of 150mg/dl and 300 mg/dl glucose. The content of test wells were the same as the control, except for different glucose concentrations. All wells were incubated for 60 minutes under the same conditions used for preincubation[27,28]. Then after gentle centrifugation, the supernatants of control and test wells were collected and kept at -20°C until insulin assay. Insulin levels were assayed by the ELISA method.

### Hydrogelic composite transplantation

The diabetic C57BL/6 recipient mice with blood glucose concentration >400 mg/dl were candidate for transplantation. Surgery by making a small incision in the abdominal wall was performed after ketamine (10 mg/kg) and xylazine (100 mg/kg) injection. The mice were divided into four groups consisting of control (without transplantation), hydrogel, HPI islets, and FGS islets, and a minimal mass of 200 islet equivalents/mouse was transplanted intraperitoneally in the recipient mice. Blood-glucose levels were monitored within a 4-day interval by glucometer (Accu-Check Performa, Germany).

### Hydrogel composite recovery and hematoxylin and eosin (H&E) staining

Mice were killed by cervical dislocation 20 days after transplantation. Composites were collected from the peritoneal cavity and washed in a saline buffer. For *in vivo* assessment of the transplanted islets, the hydrogelic grafts were recovered from each mouse, fixed in 10% formaldehyde, embedded in paraffin and cut into 5-μm sections. The sections were stained by H&E staining using a standard protocol. All grafts were examined by an independent pathologist.

### Statistical methods

Results displayed as the mean±SD of at least 6 independent experiments, each involving six mice. Tukey’s multiple comparisons test and general linear model were used to determine statistically significant difference. *P*<0.05 was considered to be statistically significant.

## RESULTS

### Comparison of islet purification by hand-picking isolation versus Ficoll gradient separation

The two islet isolation procedures were compared based on criteria such as time consumption, purity, yield, and viability of the isolated islets (Table 1). The required time for pancreas removal and islet purification was calculated for both HPI islets and FGS islets. The calculated time consumption for the HPI and FGS islet purification methods were 55.4±9.8 and 42.7±10.4 minutes, respectively. The rate of purity of the islets (%) in each method was assessed by dithizone staining and was based on the color discrimination from the acinar cells. The rates of purity for the HPI and FGS methods were 94.2±2.3 and 78.6±6.7 percent, respectively. The yield was determined as the number of islets obtained per pancreas in each method. The yield for the HPI method was 174.5±26.7, whereas the FGS method produced 136.3±12.2 islets/pancreas. To determine viability, the ratio of viable to non-viable islets or necrotic islets was calculated under an inverted microscope (4× objective lens), which was 96.2±3.2 and 82.6±4.5 for the HPI and FGS methods, respectively (Table 1).

**Table 1:** The comparison of islet purification by hand-picking isolation (HPI) versus Ficoll gradient separation (FGS) methods in BALB/c mice as donors

Criteria	Islet isolation method (mean±SD)	

	HPI	FGS
Time consumption^1^ (min)	55.4±9.8	42.7±10.4
Purity^2^ (%)	94.2±2.3	78.6±6.7
Yield^3^ (islets)	174.5±26.7	136.3±12.2
Viability^4^	96.2±3.2	82.6±4.5

1 Time consumption (min) from pancreas removal to islet purification;

2 Purity rate (%) of the islets by color discrimination from accinar cells under dithizone staining.

3 Yield is expressed as the number of islets obtained per pancreas.

4 Viability was determined as the ratio of viable to non-viable islets or necrotic islets under an inverted microscope (4× objective lens). The numbers represent the mean values±standard deviation of six independent experiments, each involving six mice.

### Insulin release from the purified murine islets

**Fig. 3:**
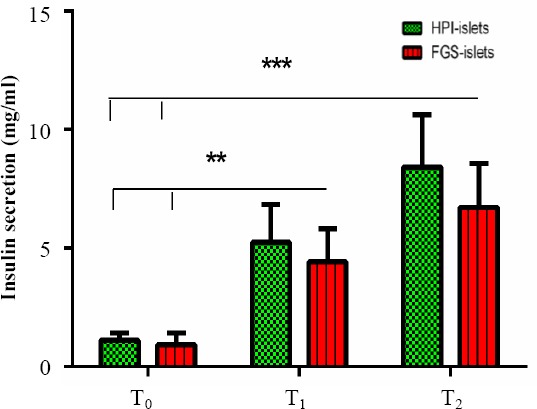
Insulin secretion from the purified islets by two procedures. Glucose-mediated insulin secretion is in equal numbers and sizes of the purified mouse islets by HPI and FGS. T0, T1, and T2 indicate the islets incubated with control medium, 150 mg/dl glucose, and 300 mg/dl glucose for 60 minutes, respectively. Data are presented as the mean±SD of six independent experiments (n=6). **P<0.01; ***P<0.001.

For functional assay of the isolated islets, glucose-mediated insulin secretion was assessed in the presence of 150 mg/dl and 300 mg/dl glucose by ELISA, and the results were compared with the control medium (Fig. 3). The results from six independent experiments showed that insulin secretion was significantly increased under different concentrations of glucose. The administration of 150 mg/dl (T1, *P*<0.01) or 300 mg/dl glucose (T2, *P*<0.001) significantly increased the insulin levels compared to the controls (T0, Fig. 3).

### Graft function

**Fig. 4:**
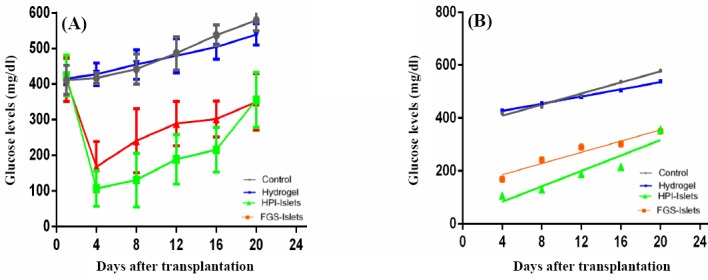
Blood glucose levels in the transplanted mice. (A) Blood glucose levels and (B) general regression in the transplanted mice. Streptozotocin-induced diabetic mice were transplanted with two pieces of 100 µl hydrogel, each containing 100 islets. Data are presented as the mean values of n=6 animals/group±SD.

The blood glucose levels of each mouse in the four defined groups were measured regularly for 20 days by a glucometer (before 9:00 A.M.). As shown in the Figure 4A, the blood-glucose levels in the control and hydrogel groups were increased rapidly without significant differences. However, in the treated groups, there was a sudden drop of glucose levels immediately after transplantation, which gradually increased. Both FGS and HPI islets maintained blood glucose levels under 400 mg/dl, for a 20-day period. For each group, a regression analysis was carried out, and the slopes (n=6 for each group) were compared by the general linear model. Differences between the slopes of the islet-embedded groups (FGS islets and HPI islets) and those without islets (control and hydrogel) were highly significant (*P*<0.0001). There were no significant differences between control and hydrogel groups or FGS islets and HPI islets groups. After islet transplantation, despite the lower blood-glucose levels in the HPI vs. the FGS-method, the difference was not statistically significant (*P*=0.08522). Graft loss was defined as the day in which the blood glucose level exceeded 400 mg/dl. Since the slopes differed greatly, it was not possible to test their statistical differences (Fig. 4B).

### Histopathology of grafts

**Fig. 5:**
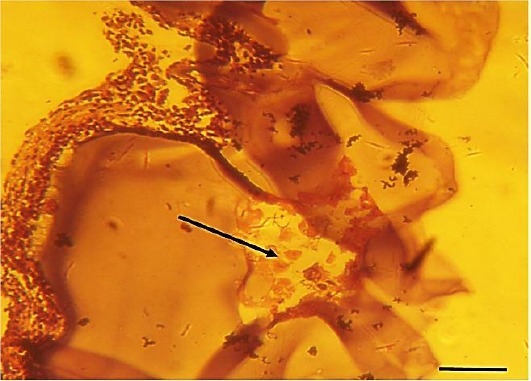
Histopathology of the grafts. The grafts were isolated after 20 days post transplantation and stained with H&E. The Figure shows a representative section with intact islets (arrow) in the hydrogel and absence of immune cell infiltration. Scale bar represents 200 μm.

The lymphocyte infiltration into or on the hydrogelic graft was assessed. H&E staining of the grafts of the transplanted groups of mice demonstrated that the immune cells were unable to infiltrate the grafts. Histologic staining also revealed that the islets remained intact in the hydrogelic graft (Fig. 5).

## DISCUSSION

Islets transplantation is an interesting experimental and a therapeutic choice for T1DM[29,30]. To overcome the requirement of large numbers of functional and viable islets in trans-plantation[31], we modified the conventional methods and developed a new one. Our model was able to resolve some of the existing transplantation difficulties.

In the conventional islet isolation techniques, large amounts of enzyme and multiple toxic density gradients of histopaque are needed. Furthermore, *in situ* pancreas perfusion through common bile duct or other applicable sites is costly and complicated and requires extensive expertise[17,22,32,33]. Some optimal gradient components, including Ficoll, Ficoll-based histopaque, dextran, and iodixanol are commonly used for purifying islets[34-36]. Li *et al*.[37] used about 5-ml collagenase type XI to digest every pancreas and injected it through common bile duct by clumping the ampulla on the duodenum wall in a microscopic manner. Similar to Carter *et al*.[32] who used three gradients of 1.109, 1.096, and 1.070, Wilson and co-workers[38] followed the digestion of purified islets by three discontinuous gradients of histopaque, including 1.108, 1.096, and 1.037. As a matter of fact, the most challenging technical step in conventional procedures is *in situ* pancreas perfusion or cannulation of the common bile duct.

In the present study, the islets were isolated via a rapid and simplified method in which *in situ* pancreas perfusion was omitted and readily carried out in a Petri dish with 1 ml of collagenase type XI using a syringe, without the need for a microscope. Then it was followed either without any gradients (HPI method) or with only two gradients of 1.077 and 1.119 histopaque (FGS method)[17]. Pancreas inflation was carried out in a short amount of time. Acinar cells synthesize and secrete up to 10 million protective and digestive enzymes in the form of inactive precursors (i.e. zymogens) and within membranous compartments (i.e. zymogen granules). Immediately after death and in the absence of protective enzymes, all digestive enzymes will be released from the acinar cells around the islets and start the islet destruction process[39-41]. However, the criteria such as time consumption, the purity rate, viability, and yield for our isolated islets were very satisfactory for transplantation or other related experiments. Although having the better yield, HPI is harder than the FGS method and requires more time.

The effects of glucose concentrations on insulin secretion were acceptable. Furthermore, the comparison of our two methods indicated that despite the long time required for the HPI purification method, its rates of purity, viability, and functional capacity of the islets make it preferable. For this method, speed and skills play very important roles.

In our opinion, due to the high quality of islets, these low cost and rapid methods could be used as alternatives to the conventional methods in all laboratories. The peritoneum cavity selected here for islet transplantation site was appropriate for our large scale hydrogelic composite containing allograft islets and more suitable for application in humans. Our model also truly demonstrated some criteria such as glucose sensing both *in vivo* and *in vitro*. Furthermore, the hydrogel is capable of improving the outcomes of the islet transplantation by facilitating the exchange of oxygen, nutrients, insulin, etc., as we showed in the experiments. Data from the *in vitro* and *in vivo* experiments on the islets resulting from the two procedures indicated that glucose was properly sensed in high concentrations, both in the hydrogel and peritoneal cavity.

Data from our *in* *vivo* study has shown that the allograft maintained the blood glucose level under 400 mg/dl (indicative of insulin secretion in the body). In general, the regression comparison demonstrated that there was a significant correlation between the transplanted islets and glucose decrease. This observation illustrated that the islets were alive and function properly *in vivo*. Due to the lack of significant differences between the HPI islets and FGS islets on glucose sensing (or insulin secretion) *in vitro* or *in vivo*, it was concluded that both of these procedures are useful in transplantation. In this model, in order to avoid autoimmune response, we embedded the allograft islets in the hydrogel. As shown in the H&E staining of the grafts, the immune cells could not infiltrate the grafts. Hence, the selected small pore size of the hydrogel prevents the passage of the immune and allograft cells, which in turn protects the graft from immune rejection or any direct cellular contact with the host.

Collectively, in the model presented here, we have introduced a low-cost and rapid method for purifying high quality islets in addition to a biomimetic hydrogel, which wraps the islets and protects them from the immune system. To improve the islets survival, we believe this model can be enhanced by adding some extracellular matrix proteins or co-transplantation with nursing cells.
